# Estimation of the Ecological Fallacy in the Geographical Analysis of the Association of Socio-Economic Deprivation and Cancer Incidence

**DOI:** 10.3390/ijerph16030296

**Published:** 2019-01-22

**Authors:** Katarina Lokar, Tina Zagar, Vesna Zadnik

**Affiliations:** 1 Institute of Oncology Ljubljana, Epidemiology and Cancer Registry, 1000 Ljubljana, Slovenia; klokar@onko-i.si (K.L.); tzagar@onko-i.si (T.Z.); 2 Faculty of Medicine, University of Ljubljana, 1000 Ljubljana, Slovenia

**Keywords:** ecological fallacy, European deprivation index, spatial units, agreement analysis, cancer incidence, public health

## Abstract

Ecological deprivation indices at the level of spatial units are often used to measure and monitor inequalities in health despite the possibility of ecological fallacy. For the purpose of this study, the European Deprivation Index (EDI) was used, which is based on Townsend theorization of relative deprivation. The Slovenian version of EDI (SI-EDI) at the aggregated level (SI-EDI-A) was calculated to the level of the national assembly polling stations. The SI-EDI was also calculated at the individual level (SI-EDI-I) by the method that represents a methodological innovation. The degree of ecological fallacy was estimated with the Receiver Operating Characteristics (ROC) curves. By calculating the area under the ROC curve, the ecological fallacy was evaluated numerically. Agreement between measuring deprivation with SI-EDI-A and SI-EDI-I was analysed by graphical methods and formal testing. The association of the socio-economic status and the cancer risk was analysed in all first cancer cases diagnosed in Slovenia at age 16 and older in the period 2011–2013. Analysis was done for each level separately, for SI-EDI-I and for SI-EDI-A. The Poisson regression model was implemented in both settings but adapted specifically for aggregated and individual data. The study clearly shows that ecological fallacy is unavoidable. However, although the association of cancer incidence and socio-economic deprivation at individual and aggregated levels was not the same for all cancer sites, the results were very similar for the majority of investigated cancer sites and especially for cancers associated with unhealthy lifestyles. The results confirm the assumptions from authors’ previous research that using the level of the national assembly polling stations would be the acceptable way to aggregate data when explaining inequalities in health in Slovenia in ecological studies.

## 1. Introduction

Ecological studies are epidemiological studies limited to the characteristics of population units or groups of people, and not individuals, or research, where exposure to risk factors is known only at the group level, while the outcomes of exposure are known at the individual level. The observed groups can be defined in different ways (according to geography, socio-economic status, type of work, etc.). Ecological variables are the characteristics of groups, organizations or spatial units, while the variables at the individual level are the characteristics of an individual [1]. Ecological studies are used to explore possible causal links between exposure to risk and specific health outcomes when other types of research are not possible or appropriate [2]. They are not used to study causal mechanisms by itself but are used to research associations. The results of such studies can set the stage for examining causal mechanisms. Ecological studies can be the best approach for exploring exposures that are easier to measure on groups of individuals than at an individual level, for example, when studying the impact of air pollution on health. Researching health determinants at this level is important. Generally, it is ideal to understand health disparities with respect to selected environmental and/or socio-demographic exposures. However, it is accompanied by numerous criticisms, which are mainly related to the transfer of results from the aggregated level to the individual level. One of the major problems of this kind of the research is ecological fallacy, when it is erroneously assumed that the statistical correlation between two variables at the aggregated level is equal to the correlation between the corresponding variables at the individual level. In extreme cases, the correlation at one level can completely disappear at another level, or can even be reversed. The higher frequency of the disease in the areas of greater exposure does not necessary mean that the exposed individuals have a greater risk of the disease than individuals who are not exposed, since no population group is completely homogeneous in terms of exposure. If each observed geographical area consisted only of inhabitants that would be exposed or only inhabitants that would not be exposed, then there would be no ecological fallacy [3,4].

Ecological research has its own advantages despite the possibility of ecological fallacy. There are several reasons for the popularity of ecological studies. They are quickly feasible and inexpensive, and they can be the best approach to exploring exposures that are easier to measure in groups than at an individual level. With them, it is possible to connect data from different (routinely managed) databases, and they are suitable for monitoring performance of public health measures at the population level. In addition, one of the most obvious reasons is open and ever-increasing availability of aggregated data. Even if individual data is available, access to them is usually blocked or at least time consuming due to the protection of privacy. Inaccessibility limits the researchers in planning their research and data analysis. Secured data are rarely accessible by the wider research community, which implicates research replication as well [4,5]. Given confidentiality concerns, often health data are aggregated to protect identify of patients. Therefore, only aggregated data are available for different health data sets.

The association between lower socio-economic status and poor effects on health has been observed in research at the individual level and in research at the ecological level [6]. Some studies have reported a closer relationship between health indicators and the socio-economic status at the individual level, while other studies have identified a similar extent of health inequalities in individual and aggregated data for the entire population or only for part of the population [7,8,9,10,11,12,13,14,15,16]. Deprivation indices are calculated values that summarize the common characteristics of several, usually routinely measured, direct indicators of socio-economic status. Mainly, due to the absence of socio-economic data of individuals in administrative databases, deprivation indices are prepared for the aggregated level. They differ among themselves depending on components they are composed of and on statistical approach used for merging these components [17]. 

The European deprivation Index (EDI) is an aggregated score of relative deprivation, which can be calculated for each European country at a small area level using for its construction data from national census and European survey specifically devoted to assess relative deprivation (European Union Statistics on Income and Living Conditions: EU-SILC). For each country, the national version of EDI is a weighted combination of census aggregated variables that are most highly correlated with the country-specific individual deprivation indicator [18]. So far, the EDI has been developed for France, Italy, Portugal, Spain, England and Slovenia [19,20,21] and has since then been used in several studies on social inequalities in cancer burdens [22,23,24], screening uptake [25] and health care access [26], orthopaedic care [27] and environmental exposure [28].

This article explores agreement of measuring socio-economic deprivation in the Slovenian population with an individual and aggregated EDI, and estimates the degree of ecological fallacy when using aggregated deprivation index in actual population-based data. The research hypothesis proposed that, when exploring the impact of socio-economic status on the cancer incidence in the Slovenian population on aggregated data, the ecological fallacy would not significantly affect the final findings. The main objective of the study was to determine whether the use of aggregated deprivation indices was appropriate for analysing socio-economic health inequalities in the Slovenian population and to evaluate the impact of ecological fallacy if the analysis was performed on the smallest spatial units for which the data were publicly available. Ideally, data must be analyzed at the smallest aggregation levels. 

The second objective was to determine with the data from the Cancer Registry of the Republic of Slovenia (CRRS) if it would be possible to adequately substitute Slovenian version of the European deprivation index at the individual level (SI-EDI-I) when exploring the association of socio-economic status and cancer incidence in the Slovenian population with the Slovenian version of the European deprivation index at the aggregated level (SI-EDI-A). As the existing methodology for EDI calculation has been developed for an aggregated level only, a supplementary aim of presented research has also been to propose an innovative expansion of EDI calculation to individual data. 

## 2. Materials and Methods 

### 2.1. Study Population and Data Sources

For the calculation of the SI-EDI-A and SI-EDI-I information from two databases was combined in this analysis: EU-SILC and the national census. In Slovenia, both databases are managed by the Statistical Office of the Republic of Slovenia (SORS) and were supplied for this research for the year 2011. The EU-SILC survey is organised by Eurostat and is based on a standardised questionnaire for interviewing a representative panel of households and individuals. It is specially designed to study deprivation and provides data on income, poverty, social exclusions and living conditions in the European Union [29]. To ensure the population was appropriately represented, all the EU-SILC responses were weighted to the survey sample design, response rate and population size for this report. There were 9247 households and 24,600 individuals aged 16 and over included in the EU-SILC survey in Slovenia in 2011. The Slovenian national census 2011 was registry-based; the existing statistical and administrative data sources were linked [30]. The census provided data on individual characteristics, features of households/families and dwellings traits for all two million Slovenian inhabitants. For the purpose of this study 1,739,865 individuals aged 16 years and older were used.

To explore the association of the socio-economic deprivation and cancer incidence data from the population-based CRRS were applied. The population of the area covered by the CRRS is the whole Slovenian population, approximately 2 million individuals. All cancer patients older than 16 years at diagnosis recorded in the CRRS between 1 January 2011 and 31 December 2013 were included in the analysis. For each cancer case, the information on patient’s gender, the permanent residence at the time of diagnosis, the date of diagnosis, the date of birth and the cancer site was used. The age of diagnosis has been calculated and categorised into 5-year age groups (15–19, 20–24, 25–29, 30–34, 35–39, 40–44, 45–49, 50–54, 55–59, 60–64, 65–69, 70–74, 75–79, 80 and older). The patients’ addresses were geolocalized using Geographic Information Systems (ArcGIS 10.4, ESRI, Redlands, CA, USA) and assigned to five different administrative geographical levels, defined by Slovenian Registry of Spatial Units of the Surveying and Mapping Authority: 3104 national assembly polling stations, 5972 settlements, 210 municipalities, 58 administrative units and 12 statistical regions. The cancer sites considered were defined according to the International Classification of Diseases—10th revision (ICD–10). Considering the statistical power, 15 solid tumour sites and two hematopoietic malignancy groups in men and women were analysed. These cancer sites were head and neck (C00–C14, C30–C32), oesophagus (C15), stomach (C16), colon and rectum (C18–C20), pancreas (C25), lung and trachea (C33–C34), melanoma of skin (C43), breast (C50), cervix (C53), uterus (C54), prostate (C61), testis (C62), kidney (C64–C65), bladder (C67), thyroid (C73), non–Hodgkin’s lymphomas (C82–C85), and leukaemias (C91–C95). They were determined by frequency of incidence, quality and completeness of collected data, and by gender relevance, using incidence data of cancer for Slovenia [31]. In addition, 27,331 cancer cases were included in the analysis. The background population data from national census in 2011 were used. The average number of the population aged 16 years and older per national assembly polling station was 561 (ranging from 28 to 4,229), per settlement 291 (ranging from 1 to 234,209), per municipality 8285 (ranging from 277 to 240,730), per administrative unit 29,998 (ranging from 7069 to 295,732) and per statistical region 144,989 (ranging from 38,122 to 448,887) respectively.

### 2.2. Ethics

In the Republic of Slovenia, individual data collected by national statistics for statistical processing are strictly confidential and any access of researchers must be in line with the National Statistics Act. For the purpose of this study, SORS has enabled the authors an access to individual data in the secure room under their supervision. SORS prepared individual microdata datasets by removing identifiers that could disclose the persons living in the observed geographical unit with high probability. SORS also enabled the access to statistically unprotected aggregated data. For this purpose and for the needs of this research, the Institute of Oncology Ljubljana and the SORS signed a Contract on the processing of personal data and access to data for scientific research purposes. 

In this research, the routinely collected individual data from the CRRS was also used. Researchers of the Institute of Oncology Ljubljana have a legal basis for the collection, processing and storage of these data (Databases Act in the area of health care, Official Gazette of RS Nos. 65/00, 47/15 and 31/18). There is no need to assure the patients’ informed consent for the purpose of epidemiological and other research as long as a patient’s identity is not identifiable. 

### 2.3. Statistical Analysis

#### 2.3.1. Calculation of the Deprivation Index

The European Deprivation Index, which was used in this study, is based on Townsend theorization of relative deprivation, which defined disadvantaged individuals as those who were not able to meet the needs identified by the majority of the people in the society in which they lived [32]. The EDI was constructed by the methodology proposed by Pornet et al. [33]. It is based on the weighted combination of geographical census variables correlated with an individual indicator of deprivation obtained from individual data from the EU-SILC. The construction of the SI-EDI consisted of the following steps:

1. Calculation of an individual deprivation indicator for persons included in the EU-SILC sample.

2. Identification of the variables that could be extrapolated from the sample to the population.

3. Calculation of the SI-EDI according to Equation (1) [21,33]:

(1) $$SI-EDI_{i}=\overset{J}{\underset{j=1}{\sum }}\beta_{j}\cdot V_{ij}$$

where *V*_*ij*_ were the basic components of SI-EDI, which were determined in the second step of the process, and *β_j_* were their regression coefficients with an individual deprivation indicator in the EU-SILC sample, which was calculated in the first step of the process. 

In the calculation of the Slovenian version of the European deprivation index at the aggregated level (SI-EDI-A), the components *V*_*ij*_ represented the proportion of individuals in the deprived category normalized to the national mean. Calculation of the Slovenian version of the European deprivation index at the individual level (SI-EDI-I) represents a methodological innovation. The SI-EDI-I deprivation score was calculated by Equation (1) [21,33] as well, but, at this point, V_ij_ values represented individual dichotomized basic components. 

The continuous version or the categorical version (national quintiles) of the SI-EDI was used alternatively according to the method used. SPSS version 24 (IBM Corp., Armonk, NY, USA) was used for the calculations described.

#### 2.3.2. Assessment of the Ecological Fallacy

The degree of ecological fallacy in the SI-EDI-A data analysis was quantified with procedure proposed by Bryere et al. [34] by using the Receiver Operating Characteristics (ROC) curve and Area Under the Curve (AUC). ROC is a probability curve and AUC represents degree or measure of separability. It tells how much of the model is capable of distinguishing between classes. With the ROC curve, the proportion of identically classified deprived individuals (sensitivity) in correspondence to identically classified affluent individuals (100-specificity) was showed for different deprivation index cut-off points. SI-EDI-I was used as a “gold standard” for comparison to SI-EDI-A measurements at the level of national assembly polling stations, where the average number of individuals per unit was 561 (with minimum 28 and maximum 4229). By calculating AUC, the ecological fallacy was evaluated numerically. The AUC was defined as the measure of how well the indices could distinguish between the deprived and the nondeprived individuals.

One of the methods to reduce the ecological fallacy is the use of the smaller spatial units, although the existence of an ecological fallacy cannot be completely avoided with this measure. This study verified this by a comparative assessment of ecological fallacy with the same procedure also for SI-EDI-A calculated at the level of settlements, municipalities, administrative units and statistical regions. The level of settlements presented in this study is the smallest spatial unit. However, in Slovenia, the city municipalities are treated as one settlement and some settlements have no inhabitants. Although there are smaller administrative spatial units available in Slovenia, there is an issue with data protection. The statistically unprotected aggregated data cannot be taken out of SORS’ secure room when there is a risk of disclosure. Risk of disclosure is assessed according to the rules for determining sensitive statistics, and the threshold for aggregated data in this study was at least 30 individuals per spatial unit. For example, the smallest Slovenian spatial units are at the level of spatial district. There are 18,037 spatial districts and, in 2011, there were 4649 spatial districts with less than 30 individuals, of which 1005 districts had no inhabitants. Prior research by the authors indicated that using the level of the national assembly polling stations would be an acceptable and feasible way to aggregate data for analysing the association of socio-economic factors and cancer risk [21]. Firstly, it would meet the demands of individual data protection, as in 2011 there were only seven national assembly polling stations with less than 30 individuals and this problem could be solved by merging this units with their neighboring units. Secondly, it would merge the population of city settlements at smaller spatial units. Thirdly, the law on the national assembly elections regulates that national assembly election spatial units, such as constituencies and electoral districts and consequently polling stations, are drawn up by taking into account geographical, cultural and other common features (The Law on the National Assembly Elections, Official Gazette of RS, no. 109/06 - official consolidated text, 54/07 - odl. US and 23/17), which other administrative areas do not necessarily follow.

SPSS version 24 (IBM Corp., Armonk, NY, USA) was used for the procedures described.

#### 2.3.3. Measuring Agreement Analysis

SI-EDI-A and SI-EDI-I represent two measures of deprivation. The degree of agreement between them was analysed applying statistical procedures that have traditionally been used for assessing agreement between two methods of clinical measurement. The descriptive assessment of agreement of SI-EDI-A and SI-EDI-I measurements was based on two graphical methods and on formal testing:

1. Bland–Altman plot or Difference plot [35,36] is a scatter plot in which the difference between the paired measurements (A-B) is plotted against their mean value [(A+B)/2]. In this study, the A represents SI-EDI-I and B represents SI-EDI-A for polling stations. The graph provides two main pieces of information, namely the average of all the differences, which is often called the bias (a measure of accuracy), and the 95% limits of agreement (a measure of precision). Accurate measurements are close to the true value, irrespective of the spread of the measurements. An ideal model would claim that the measurements obtained by one method or another gave exactly the same results. Thus, all the differences would be equal to zero. In contrast, precise measurements are close to each other, irrespective of their deviation from the true value [37,38]. The differences between the paired measurements should be normally distributed. This was tested in a presented study graphically by normal quantile–quantile (QQ) plot and numerically by Kolmogorov–Smirnov test.

2. Mountain plot or “folded empirical cumulative distribution plot” [39] is a display of percentiles for each ranked difference between all pairs of measurements. By estimating the deviation of the top of the curve from zero, a systematic error (bias) was evaluated.

3. The index of agreement was calculated with Lin’s coefficient [40] or concordance correlation coefficient (CCC) as an index of reliability:

(2) $$\rho_{c}=\frac{2\rho \sigma_{X}\sigma_{Y}}{{\left(\mu_{X}-\mu_{Y}\right)}^{2}+\;\sigma_{X}^{2}\;+\;\sigma_{Y}^{2}}$$

where *μ* was the expected value, *σ* was the standard deviation, *ρ* was correlation (the Pearson correlation coefficient), and the *x* and *y* indicate both compared method (SI-EDI-A and SI-EDI-I). The Lin’s coefficient represents the expected square difference of the pair of measurements from the equality line (*x* = *y*), adapted to lie between –1 and 1, where 1 means complete concordance. 

For this part of statistical analysis, the R-studio packages “BlandAltmanLeh”, “mountainplot” and “DescTools” (RStudio version 1.0.153, RStudio, Inc., Boston, MA, USA) and SPSS version 24 (IBM Corp., Armonk, NY, USA) were used.

#### 2.3.4. Analysis of the Association of Socio-Economic Deprivation and Cancer Incidence

For this part of the analysis, the CRRS database was joined with the continuous versions of SI-EDI-I and SI-EDI-A on the level of polling stations. The association between the socio-economic status and the cancer incidence was modelled under the assumption that the number of observed cancer cases fits Poisson’s distribution. Poisson regression is used for variables where results only have positive values, such as counted events and rates. They often follow the Poisson distribution and can therefore be used in the Poisson regression model. The method is widely used in epidemiology and is especially useful for rare diseases and large populations, such as when the number of cases of disease is low compared to the total size of the study population [41,42,43]. 

The values of the regression coefficients for the individual level were calculated by the classical Poisson regression model. The analysis was done by SPSS version 24 (IBM Corp., Armonk, NY, USA). The regression coefficient associated with the variable SI-EDI-I and its 95% confidence interval was estimated. A positive parameter related to SI-EDI-I is interpreted as a relatively higher incidence in disadvantaged individuals, and a negative parameter related to SI-EDI-I is interpreted as relatively higher incidence in affluent individuals. 

For the aggregated level, a Bayesian approach was used rather than a classical Poisson regression because of expected occurrence of overdispersion defined as variability in the number of cases to be higher than expected by the Poisson distribution. The differences in population sizes of the polling station units, called unstructured spatial heterogeneity, might introduce variations and this method permits the distinction between random fluctuations and true variations in the incidence rates. Moreover, neighbouring areas may not be independent and have similar incidence rates. This is called spatial autocorrelation and is also integrated in the Bayesian approach [23,44]. Therefore, the overdispersed Poisson model was expanded by including spatially dependent and spatially independent random variables and treated with the Bayesian approach using WinBUGS version 1.4 (MRC and Imperial College of Science, Technology and Medicine, Cambridge and London, UK) [45]. The following hierarchical convolutional Bayes model [46] was used:

(3) $$\ln\frac{O_{i}}{E_{i}}={\mathrm{lnE}}_{i}+a+\overset{J}{\underset{j=1}{\sum }}\beta_{j}X_{\mathrm{ij}}+H_{i}+S_{i}$$

where a represented the basic (logarithmic) relative risk of disease in the entire study area. O_i_ and E_i_ represented the observed and expected number of cases in the i-th polling station. X_ij_ was a set of J explanatory variables for an individual polling station that was empirically obtained (in our case SI-EDI-A). β_j_ were the regression coefficients for the j-th explanatory variable. H_i_ were unstructured (heterogeneous) random factors that were geographically independent and S_i_ was a spatially dependent component (spatially structured heterogeneity). It was defined by a conditionally autoregressive (CAR) prior probability distribution. The regression coefficient associated with the variable SI-EDI-A and its 95% confidence interval was estimated. A positive parameter related to SI-EDI-A means an over-incidence in disadvantage areas and a negative parameter related to SI-EDI-A means an over-incidence in affluent areas. 

## 3. Results

The results are presented for each step of the analysis separately.

### 3.1. The Slovenian Version of the European Deprivation Index

The SI-EDI-A has been calculated on five geographical levels: national assembly polling stations, settlements, municipalities, administrative units and statistical regions. Table 1 presents the comparison of the SI-EDI-A across all five levels. The results for SI-EDI-I are appended as well. All individuals and all spatial units at different geographical levels were classified according to its level of deprivation using the quintiles of the SI-EDI score as cut-offs. The proportion of comprised study population in each quintile at each geographical level is also shown in Table 1. The first quintile represents least deprived and the fifth quintile most deprived population.

### 3.2. Assessment of the Ecological Fallacy

**Figure 1:**
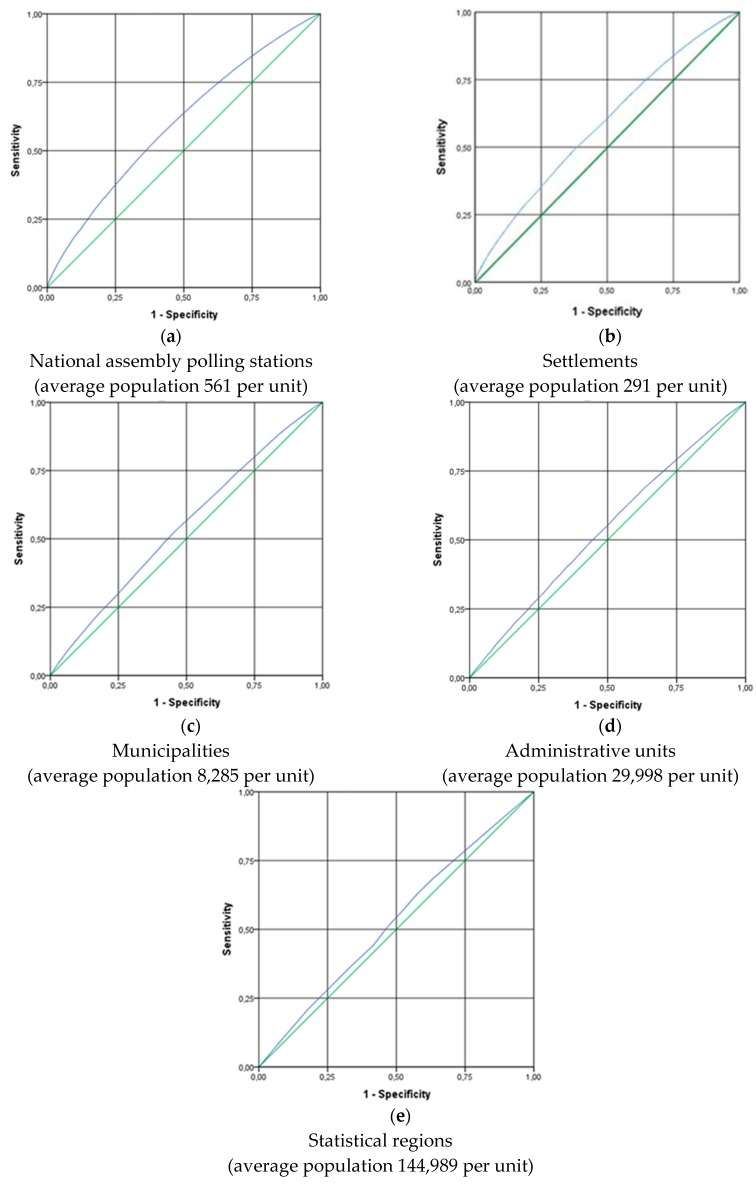
ROC curves of ecological indices at different levels of aggregation according to individual deprivation (aggregated and individual data from population census in 2011): (**a**) SI-EDI-A for national assembly polling stations; (**b**) SI-EDI-A for settlements; (**c**) SI-EDI-A for municipalities; (**d**) SI-EDI-A for administrative units; (**e**) SI-EDI-A for statistical regions. In an ROC curve, the true positive rate (Sensitivity) is plotted in a function of the false positive rate (100-Specificity) for different cut-off points of a deprivation index. Each point on the ROC curve represents a different cut off value. The closer the curve follows the left-hand border and then the top border of the ROC space, the more performant the deprivation index. The closer the curve comes to the 45-degree diagonal of the ROC space, the less performant the deprivation index.

ROC curves were constructed to evaluate the sensitivity and specificity of SI-EDI-A deprivation indices at the level of the national assembly polling stations, settlements, municipalities, administrative units, and statistical regions, using individual SI-EDI-I deprivation index as defined above as the gold standard (Figure 1).

This analysis showed that no index at an aggregated level was highly performant at discriminating between affluent and deprived subjects at an individual level. The ecological deprivation indices at municipalities, administrative units and statistical regions level had an ROC curve that was closer to the diagonal, suggesting that it is less adapted to capture individual deprivation. Between the cut off point with a sensitivity of 25% and specificity of 75%, the indices at national assembly polling stations level and at settlements level seemed much more performant.

In accordance with the ROC curves, the AUC values and their confidence intervals (Table 2) indicate that the indices of municipalities, administrative units, and statistical regions showed significantly weaker performance than the national assembly polling stations and settlements indices, with a value of AUC close to the 0.5, a value that is equivalent to chance. The national assembly polling stations index had a value of 0.600, which suggests a 60% chance to correctly distinguish between affluent and deprived subjects at an individual level. 

Distribution of deprived and non-deprived individuals (according to the SI-EDI-I) into the different quintiles of the ecological deprivation indices (SI-EDI-A) is shown in Table 3 and Table 4. Approximately 30% (ranging from 24.1% to 36.8%) of disadvantaged individuals according to SI-EDI-I was distributed to the quintile 5 (most deprived) of the ecological deprivation indices. Between 8.5% and 14.1% of disadvantaged individuals were distributed to the most affluent category (quintile 1) of the ecological deprivation indices. 

Only between 10 to 22% of non-deprived individuals according to SI-EDI-I were distributed to the quintile 1 (most affluent) of the ecological deprivation indices and a little higher percentage to the quintile 2. Analysing the distribution of the deprived and non-deprived individuals according to SI-EDI-I showed that ecological deprivation indices (SI-EDI-A) do not distinguish deprived and affluent individuals in the same manner, but the indices capture deprivation better than affluence.

### 3.3. Agreement Analysis

**Figure 2:**
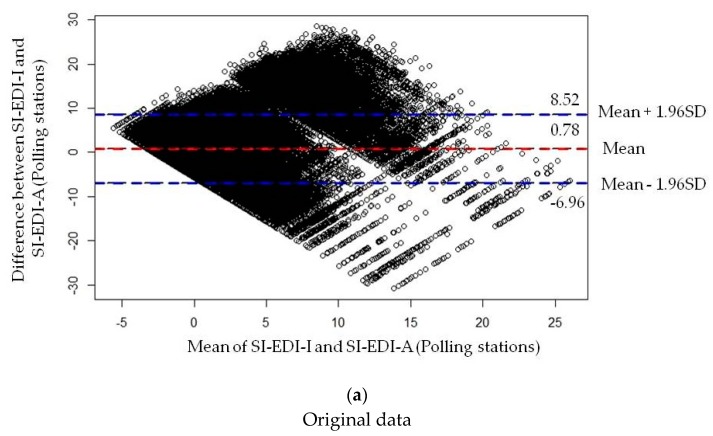

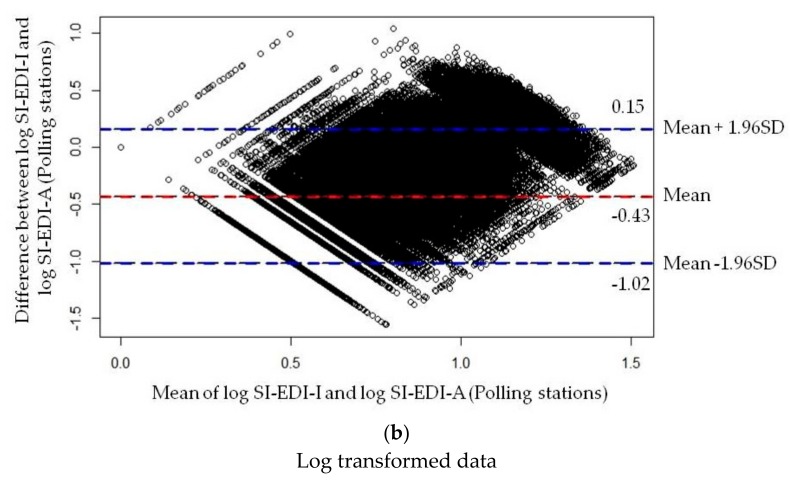
Bland–Altman plot: (**a**) Bland–Altman plot of original data; (**b**) Bland–Altman plot of logarithmic transformation of the data.

Figure 2a shows the Bland–Altman plot where the differences between the paired measurements of SI-EDI-I and SI-EDI-A for polling stations were plotted on the ordinate. The normal quantile–quantile plot showed that the assumption of normality may not be valid for this variable. This was also confirmed by the Kolmogorov–Smirnov test where the *p*-value was lower than 0.05, so the hypothesis of normality was rejected. The most common departure from the assumptions that the mean and standard deviation of the differences are the same throughout the range of measurement is an increase in variability of the differences as the magnitude of the measurement increases. In such cases, a plot of one method against the other shows a spreading out of the data for larger measurements, which was the case with this data. In Figure 2a, how the differences increase as the SI-EDI score rises can be seen.

Under these circumstances, logarithmic (log) transformation of both measurements before analysis will enable the standard approach of the Bland–Altman method to be used [36]. Following this recommendation, logarithmic transformation of SI-EDI-I and SI-EDI-A for polling stations scores was performed. In the presented case, the log transformation was only slightly successful in producing differences unrelated to the mean. Figure 2b shows the log transformed data and the difference versus mean plot with 95% limits of agreement. The mean difference (log SI-EDI-I – log SI-EDI-A) is –0.43 with 95% limits of agreement –1.02 and 0.15. In favour of the interpretation, the back-transform (antilog) of the results has been done. In the original data in Figure 2a, the mean difference (SI-EDI-I – SI-EDI-A) is 0.78 with 95% limits of agreement –6.96 and 8.52. SI-EDI-I scores may be lower for 6.7 or higher for 8.5 compared to SI-EDI-A scores for polling stations and, on average, SI-EDI-I measures deprivation scores higher for 0.78 (Figure 2a). Both Bland–Altman plots reflect the discrepancy between the methods, as many points lie outside the limits of agreement. 

**Figure 3:**
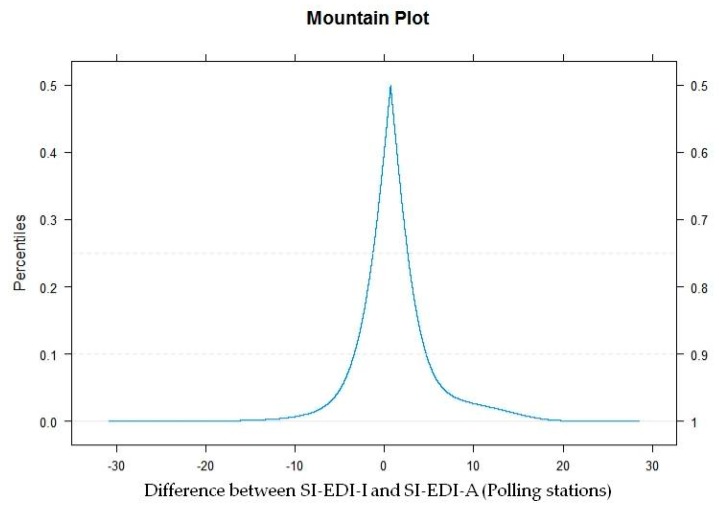
Mountain plot.

The Mountain plot shown in Figure 3 presents the same discrepancy as long tails in the plot reflect large differences between the methods. Finally, the same pattern was found for the CCC (Lin’s coefficient): 0.1935 with 95% CI = 0.1922; 0.1950. For good agreement between the methods, a CCC closer to 1 would be expected.

### 3.4. Association of Socio-Economic Deprivation and Cancer Incidence

A total of 27,331 cancer cases categorised into 17 cancer sites were analysed. Table 5 presents the combined results of a classical Poisson regression analysis using the continuous version of the SI-EDI-I and the results of Bayesian Poisson modelling using the continuous version of the SI-EDI-A for polling stations.

A statistically significant higher incidence among the disadvantaged at the individual level was observed for cancers of the head and neck, oesophagus, stomach, colon and rectum, pancreas, lung and trachea, uterus and bladder. A significantly higher incidence in affluent individuals was observed for melanoma, prostate and thyroid cancer. At the aggregated level, a statistically significant higher incidence among the disadvantaged was observed for cancers of the head and neck, oesophagus and lung, and trachea. A significantly higher incidence among affluent individuals was observed for melanoma, breast and prostate cancer. Stronger association of socio-economic deprivation and cancer incidence can be observed at the individual level. 

By comparing the direction and magnitude of the SI-EDI-I and SI-EDI-A coefficients, statistically significant results can be observed for head and neck, oesophagus, lung and trachea, melanoma and prostate cancer at the individual and aggregated level. Stomach, colon and rectum, pancreas, uterus, bladder and thyroid cancer had statistically significant results only at the individual level, while breast cancer had a statistically significant result only at the aggregated level. No statistically significant results at either level were observed for cervix, testicular, kidney cancers and for Non-Hodgkin’s lymphomas and leukaemia. When comparing results at both levels also in terms of the overlapping confidence intervals, altogether fifteen out of seventeen comparisons agreed. A paired-samples *t*-test was conducted to compare SI-EDI-I and SI-EDI-A coefficients. There was not a significant difference in the SI-EDI-I (Mean = 0.01, SD = 0.04) and SI-EDI-A (Mean = −0.001, SD = 0.02) coefficients; t(16) = 1.48, *p* = 0.157.

## 4. Discussion

The aim of this article was to reveal whether the use of aggregated deprivation indices was appropriate for analysing socio-economic health inequalities in the Slovenian population and to evaluate the impact of ecological fallacy if the analysis was performed on the smallest possible spatial units for which the data were publicly available. On one side, the ecological fallacy has been determined by different graphical and numerical methods, but, on the other side, the association of socio-economic inequalities and the cancer burden at individual and aggregated levels when compared was affected by ecological fallacy in only two out of seventeen studied cancer sites and the formal test of differences showed no statistically significant difference.

The ecological fallacy was assessed by the ROC curves and by calculation of the area under the curves. Using different levels of spatial units, this research demonstrated change in the degree of ecological fallacy with the increase in aggregation level. As is evident, the confidence interval shrank as the aggregation level increased. The results also showed that none of the five aggregated deprivation indices (SI-EDI-A at the level of the national assembly polling stations, settlements, municipalities, administrative units, and statistical regions) was clearly better than the others. SI-EDI-A at the level of the national assembly polling stations that were used as the smallest available spatial units in this study performed better than the other indices in particular between the cut off point with a sensitivity of 25% and specificity of 75%. Although the level of settlements is smaller than the level of national assembly polling stations, SI-EDI-A for settlements showed weaker performance. The anomaly that can be seen at the level of settlements is explainable by the fact that, in Slovenia, the city municipalities are treated as one settlement, for example the capital of Slovenia has a population of almost 300,000. This outcome also confirms assumptions from the authors’ previous research that indicated that using the level of the national assembly polling stations as the smallest spatial units would be acceptable way to aggregate data [21].

Ecological fallacy is unavoidable when assessing deprivation using aggregate indices, even when very small spatial units are used. One way to improve the performance of aggregated deprivation indices would be to redefine the boundaries of the applied geographic areas. Namely, the administrative boundaries do not necessarily coincide with neighbourhood boundaries as perceived by inhabitants. However, it appears impossible to avoid using administrative boundaries because they are the only way to define the background population using census data [34]. In cases when individual data are available, individual indices can be used and aggregation is not needed.

To measure agreement between deprivation index at the individual level (SI-EDI-I) and deprivation index at the ecological level (SI-EDI-A), the Bland–Altman method was used, an approach that has been routinely used in clinical settings. Medical laboratories often need to assess the agreement between two measurement methods. Every time they have to change one method for another one, or evaluate a new or alternative method, or have an alignment problem between two instruments, they need some tools to measure and appraise the differences as well as the cause of these differences. Validation of a clinical measurement should include all of the procedures to demonstrate that a particular method used for the quantitative measurement of the variable concerned is both reliable and reproducible for the intended use. The measurement of variables always implies some degree of error. When two methods are compared, neither provides an unequivocally correct measurement, so it could be interesting trying to assess the degree of agreement [38]. To assess this degree of agreement, the correct statistical approach is not obvious. Many studies give the product–moment correlation coefficient (r) between the results of two measurement methods as an indicator of agreement. However, correlation studies the relationship between two variables, not the differences, and it is not recommended as a method for assessing the comparability between methods [35,36,38]. In 1983, Altman and Bland proposed an alternative analysis based on the quantification of the agreement between two quantitative measurements by studying the mean difference and constructing limits of agreement [47]. With the extensive literature search, the authors could not find the study or an example where such approach had been used before in a similar setting to this study. They also did not find any indication as to why it was not advisable to use it in the presented case.

An important problem is to assess how narrow the limits of agreement need to be before two techniques can be considered interchangeable [48]. The Bland–Altman plot does not say if the agreement is sufficient or suitable to use one method or the other indifferently. It simply quantifies the bias and a range of agreement, within which 95% of the differences between one measurement and the other are included. For example, it is possible to say that the bias is significant because the line of equality is not within the confidence interval of the mean difference, but only analytical, biological or clinical goals could define whether the agreement interval is too wide or sufficiently narrow for chosen purpose. The best way to use the Bland–Altman plot would be to define a priori the limits of maximum acceptable differences (limits of agreement expected), based on biologically and analytically relevant criteria, and then to obtain the statistics to see if these limits are exceeded, or not [35,36,38]. Bland–Altman plots (Figure 2) from this study reflect the discrepancy between the SI-EDI-I and SI-EDI-A. The question here, which was not possible to answer at this time, was what would be acceptable differences between studied methods of measuring deprivation index? What would be relevant criteria to define whether the agreement interval is too wide or sufficiently narrow for chosen purposes? This methodological challenge in the application of the Bland–Altman method should be explored further in future research.

Poor statistical agreement of SI-EDI-I and SI-EDI-A is not reflected in the results that describe the socio-economic inequalities in the cancer burden at the individual level or aggregated analysis. Irrespective of the type of deprivation indicator (aggregated or individual), in Slovenia, people further down the social ladder are at a higher risk of head and neck, oesophageal and lung cancers than those closer to the top. On the other hand, the risk of malignant melanoma and prostate cancer is higher in affluent populations. When comparing results from this study to the results of other studies [23,49] that applied EDI as a deprivation index a statistically significant higher incidence among disadvantaged for head and neck, oesophagus (in other studies only male) and lung and trachea, and a statistically significant higher incidence among affluent for melanoma and prostate cancer can be observed in all compared results. The sites identified as linked with socioeconomic status are not surprising, and all these cancers can be explained by higher risk behaviours. In all compared results, no correlation to socioeconomic status was found for kidney, non-Hodgkin’s lymphomas and leukaemias. The results in this study for individual level showed more similarities with other two studies [23,49] compared to the results for the aggregated level. One of the reasons can be a much higher number of cancer cases in comparable studies due to larger populations. 

By estimating the burden of social deprivation on cancer incidence with a site-specific approach, it is possible to better identify the kinds of public health measures appropriate for reducing social inequalities. The use of geographical approaches with relevant deprivation indices also allows better identification of populations that should be targeted in such prevention efforts [23]. 

The whole research presented in this article is based on European deprivation index (EDI). The EDI is an ecological deprivation index, which summarises the socioeconomic status of individuals according to the level of deprivation assigned to the geographical area they live in [33]. The weights are attributed according to the role (association) each variable had in predicting individual deprivation. Most of other available indicators are simply the unweighted sum of variables pragmatically chosen from the census; weighting is rare and usually justified by statistical criteria only [20]. The development of the EDI was grounded on a solid theoretical framework, used both individual and aggregate variables, and relied on a longitudinal Europe-wide survey (EU-SILC) that guarantee EDI can be replicated over the time and in any of the EU member states [21]. EU-SILC has been specifically designed to measure and monitor poverty and deprivation across the EU territory, which further legitimizes the indicator. 

Ecological deprivation indices used as a ‘proxy’ of individual socioeconomic status could mistakenly classify individuals into wrong deprivation category [33]. Using the smallest available spatial units decreases the ecological fallacy because the accuracy of socioeconomic measures decreases with the size of the geographical unit used [50,51]. Pornet et al. [33] argue that the methodology proposed for EDI development should minimise ecological fallacy in comparison to techniques used to construct indices based on census, principal component analysis or factor analysis in which correlations between ecological variables are analysed without reference to the individual socioeconomic status. Moreover, from a methodological point of view, the EDI could be calculated for very small geographical areas with small populations, the only scale restriction being the area level for which the census data are available [33].

The EDI has so far been developed in seven countries, always at an aggregated level, for the smallest spatial unit in the country for which census data is routinely available [19,20,21]. The development of the EDI at the individual level for the purpose of this study thus represents a methodological innovation. The main advantage of the proposed SI-EDI-I is that it is defined by the same variables that were used in ecological SI-EDI-A and as such best reflects deprivation both at the individual and at the ecological level. The study shows that the proposed calculation of the deprivation index is appropriate for both aggregated and individual data.

Lalloué proposed creating socioeconomic categories instead of defining deprivation quintiles using hierarchical clustering that provides categories with more homogeneous compositions [52]. Part of this study was to evaluate the extent to which the deprivation index at the aggregated level can be considered as an acceptable “proxy” of individual deprivation. However, the relevance of ecological deprivation indices is viewed broader than this because they also integrate the potential effect of the neighbourhood on the individual deprivation. The multilevel studies are becoming more relevant than studies based only on individual data because they may induce an atomistic fallacy that occurs by drawing inferences regarding variability across groups [34,53]. Thus, the question of whether it is better to use the individual or aggregated deprivation index for analyzing socio-economic health inequalities in the Slovenian population remains open as more research is needed.

Lastly, Table 3 shows that 13% of deprived individuals at the polling station level live in the least-deprived quintile. This can be an indication that social segregation and stratification in Slovenia is low. The World Factbook [54] ranks Slovenia 153 out of 157 countries in terms of the GINI coefficient with GINI index of 24.4. This index measures the degree of inequality in the distribution of family income in a country. The more nearly equal a country’s income distribution, the lower its Gini index and the more unequal a country’s income distribution, the higher its Gini index. If income were distributed with perfect equality, the index would be zero; if income were distributed with perfect inequality, the index would be 100. In conclusion, the ecologic fallacy in most of the world should be less than what was found in this study. 

## 5. Conclusions

This article demonstrates the effects of aggregation on the degree of ecological fallacy using deprivation index and incidence of cancer. Ecological fallacy arises when data are aggregated, which often results in loss of details and generalization. Ecological fallacy was extensively explored from different aspects. The quantification of the ecological fallacy in this study was done with an actual population-based data set that represents an important advance in understanding of the behavior of real-world data sets in this context. 

The finding of this study warrants researchers’ attention to the degree of ecological fallacies in epidemiological studies that rely on aggregated data sets.

As part of this research, the European deprivation index at the individual level was developed. Deprivation index at the individual level played a key role in assessment of the ecological fallacy, which is inherent to the deprivation index at an aggregated level. The expansion of EDI calculation to individual data showed that calculation of the EDI is appropriate for both aggregated and individual data. 

When exploring the association of socio-economic status and the cancer incidence in the Slovenian population on aggregated data, the ecological fallacy does not significantly affect the final findings. The use of aggregated deprivation indices at the smallest spatial units for which the data are publicly available is appropriate for analysing socio-economic health inequalities in the Slovenian population. Comparison of the SI-EDI-I and SI-EDI-A coefficients showed a sufficient agreement. It is possible to adequately substitute SI-EDI-I when exploring the association of socio-economic status and cancer incidence in the Slovenian population with the SI-EDI-A at the level of national assembly polling stations.

The study also confirms previous research findings that there are certain types of cancer in Slovenia, which are more common in the socio-economically more deprived population, and others whose risk is greater in the socio-economically more affluent population.

The presented research provides to the Slovenian public health profession a key insight into the appropriateness of the methods used to assess health inequalities due to differences in the socio-economic status. It confirms that using the level of the national assembly polling stations would be the appropriate way to aggregate data when explaining inequalities in health in Slovenia in ecological studies. Although the association of cancer incidence and socio-economic deprivation at individual and aggregated levels was not the same for all cancer sites, the results were similar for the majority of investigated cancer sites, especially for cancers associated with unhealthy lifestyles.

## Figures and Tables

**Table 1:** The comparison of SI-EDI across different levels.

Level	SI-EDI-I	SI-EDI-A				
	Individual	Polling Station	Settlement	Municipality	Administrative Unit	Statistical Region
Number of units	1,739,865	3104	5972	210	58	12
% of study population in Quintile 1 ^1^	16.8%	23.2%	20.0%	15.6%	36.8%	35.6%
% of study population in Quintile 2 (cut off)	22.8% (20%: −2.12)	22.4% (20%: −2.86)	19.9% (20%: −2.05)	21.5% (20%: −2.90)	22.0% (20%: −3.50)	14.9% (20%: −3.84)
% of study population in Quintile 3 (cut off)	20.0% (40%: −1.08)	22.6% (40%: −1.52)	7.0% (40%: −0.98)	43.2% (40%: −1.37)	13.5% (40%: −1.23)	15.2% (40%: −0.31)
% of study population in Quintile 4 (cut off)	20.1% (60%: −0.29)	19.5% (60%: −0.01)	33.0% (60%: −0.68)	13.2% (60%: 0.26)	15.9% (60%: 0.67)	25.0% (60%: 0.86)
% of study population in Quintile 5 (cut off) ^2^	20.3% (80%: 1.37)	12.3% (80%: 2.32)	20.0% (80%: 0.03)	6.4% (80%: 2.41)	11.8% (80%: 3.36)	9.3% (80%: 3.85)

^1^ Least deprived. ^2^ Most deprived.

**Table 2:** Areas under curves (AUC) with 95% confidence interval of the receiver operating characteristics of ecological deprivation indices compared to individual deprivation index (aggregated and individual data from population census in 2011).

Spatial Units (SI-EDI-A Levels)	Individual Deprivation (SI-EDI-I)
SI-EDI-A for national assembly polling stations	0.600 [0.598, 0.601]
SI-EDI-A for settlements	0.585 [0.584; 0.586]
SI-EDI-A for municipalities	0.547 [0.546; 0.549]
SI-EDI-A for administrative units	0.538 [0.537; 0.539]
SI-EDI-A for statistical regions	0.530 [0.529; 0.531]

**Table 3:** Distribution of deprived people (*n* = 353,433) according to the SI-EDI-I (quintile 5) into the different quintiles of the ecological deprivation indices (SI-EDI-A).

Category/Index	SI-EDI-A for National Assembly Polling Stations	SI-EDI-A for Settlements	SI-EDI-A for Municipalities	SI-EDI-A for Administrative Units	SI-EDI-A for Statistical Regions
Quintile 1	13.0%	13.5%	14.1%	11.2%	8.5%
Quintile 2	16.6%	17.9%	19.3%	23.4%	23.0%
Quintile 3	19.2%	7.2%	20.3%	20.1%	18.4%
Quintile 4	22.3%	33.7%	17.2%	21.2%	13.3%
Quintile 5	29.0%	27.7%	29.2%	24.1%	36.8%

**Table 4:** Distribution of non-deprived people (quintiles 1 to 4, *n* = 1,386,432) according to the SI-EDI-I into the different quintiles of the ecological deprivation indices (SI-EDI-A).

Category/Index	SI-EDI-A for National Assembly Polling Stations	SI-EDI-A for Settlements	SI-EDI-A for Municipalities	SI-EDI-A for Administrative Units	SI-EDI-A for Statistical Regions
Quintile 1	21.8%	21.7%	18.7%	14.1%	10.1%
Quintile 2	20.9%	20.5%	20.5%	26.0%	26.5%
Quintile 3	20.2%	7.0%	21.0%	19.8%	17.2%
Quintile 4	19.4%	32.9%	15.6%	19.7%	12.4%
Quintile 5	17.8%	18.0%	24.3%	20.5%	33.7%

**Table 5:** Association of socio-economic deprivation at individual and aggregated level and cancer incidence in Slovenia between 2011 and 2013.

Sites	Topography (ICD−10)	Total Number of Cancer Cases	Estimation of SI-EDI-I Coefficient ^1^	95% CI ^3^	Estimation of SI-EDI-A ^2^	95% CI ^3^
Head and neck	C00–C14, C30–C32	1278	**0.071**	**0.060 to 0.082**	**0.021**	**0.001 to 0.021**
Oesophagus	C15	241	**0.056**	**0.031 to 0.082**	**0.061**	**0.022 to 0.096**
Stomach	C16	1396	**0.033**	**0.022 to 0.044**	0.009	−0.011 to 0.027
Colon and rectum	C18–C20	4398	**0.028**	**0.021 to 0.034**	−0.004	−0.015 to 0.006
Pancreas	C25	1030	**0.038**	**0.026 to 0.051**	−0.002	−0.025 to 0.021
Lung and trachea	C33–C34	3647	**0.038**	**0.031 to 0.045**	**0.019**	**0.007 to 0.031**
Skin, melanoma	C43	1471	**−0.054**	**−0.076 to −0.032**	**−0.021**	**−0.042 to −0.800**
Breast	C50	3692	−0.003	−0.012 to 0.007	**−0.018**	**−0.031 to −0.005**
Cervix	C53	377	0.022	−0.005 to 0.049	−0.001	−0.036 to 0.033
Uterus	C54	928	**0.033**	**0.019 to 0.046**	−0.020	−0.045 to 0.003
Prostate	C61	4418	**−0.014**	**−0.022 to −0.006**	**−0.017**	**−0.029 to −0.004**
Testis	C62	302	−0.050	−0.105 to 0.004	0.003	−0.035 to 0.041
Kidney	C64–C65	1057	0.006	−0.010 to 0.023	−0.011	−0.034 to 0.011
Bladder	C67	926	**0.022**	**0.008 to 0.036**	0.002	−0.023 to 0.026
Thyroid	C73	431	**−0.075**	**−0.112 to −0.038**	−0.016	−0.052 to 0.018
Non−Hodgkin’s lymphomas	C82−C85	1035	0.012	−0.002 to 0.027	−0.015	−0.038 to 0.007
Leukaemias	C91−C95	704	0.013	−0.004 to 0.031	−0.012	−0.040 to 0.015

^1^ Estimation of the coefficient related to SI-EDI-I. Positive for over-incidence in deprived individuals, negative otherwise. ^2^ Estimation of the coefficient related to SI-EDI-A for polling stations. Positive for over-incidence in deprived areas, negative otherwise. ^3^ 95% confidence interval (CI), significant results are in bold type.
